# Supplementary material for: EnzML: multi-label prediction of enzyme classes using InterPro signatures
Source: BMC Bioinformatics. 2012 Apr 25;13:61. doi: 10.1186/1471-2105-13-61 (PMC3483700; doi:10.1186/1471-2105-13-61)
Supplement: Addtional file 5 — The Java code to format the data files, evaluate and predict. The file enzml_java_code.tar.gz contains the Java code used to format database data to ARFF and XML formats, to execute cross and train-test (jackknife) evaluations and to record evaluation results to database. More information is included in the readme.txt file and the Javadoc files. The code can be used with a MySQL database. To use a different database software, other JDBC drivers might be required. [file 1471-2105-13-61-S5.gz › java_code/utils/doc/uk/ac/ed/inf/utils/MapUtils.html]

MapUtils


---


|  |  |  |
| --- | --- | --- |
| **Overview** \| **Package** \| **Class** \| **Use** \| **Tree** \| **Deprecated** \| **Index** \| **Help** | |  |
| **PREV CLASS**   **NEXT CLASS** | **FRAMES**    **NO FRAMES**     **All Classes** |  |
| SUMMARY: NESTED \| FIELD \| CONSTR \| METHOD | DETAIL: FIELD \| CONSTR \| METHOD |  |


---


## uk.ac.ed.inf.utils Class MapUtils

```
java.lang.Object
  uk.ac.ed.inf.utils.MapUtils
```

---

``` public class MapUtils extends java.lang.Object ```

Utilities to manipulate maps and hashmaps

**Version:**
:   4 Jan 2008

**Author:**
:   Luna De Ferrari luna.deferrari-at-ed.ac.uk

---

**Constructor Summary**

`MapUtils()`


| **Method Summary** | |
| --- | --- |
| `static java.util.Map.Entry` | `extractMapEntry(java.util.Map map, int i)`             Extracts a random entry (key-value association) from a map |
| `static java.util.TreeMap<java.lang.String,java.lang.String>` | `fromTwoVectorsToMap(java.util.Vector<java.lang.String> keys, java.util.Vector<java.lang.String> values)`             Creates an hashmap from 2 vectors. |
| `static double` | `getDoubleFromHashmap(java.util.HashMap hashMap, java.lang.String hashMapKey)`             Gets a double from a hashMap |
| `static java.util.ArrayList<java.lang.String>` | `getFirstValueOccurrence(java.util.Map<java.lang.String,java.lang.String> map, java.lang.String value)`             Get all the keys that have the given value in a Map\ |
| `static java.util.Map<java.lang.Integer,java.util.Map.Entry>` | `getIndexedMap(java.lang.Object[] mapEntries)`             Get a map with entries composed of: an integer id as key and an entry as value |
| `static java.lang.String` | `mapToString(java.util.Map map)`             Creates a string representation of a map, as index,tab,key,tab,value |
| `static void` | `printMap(java.util.Map map)`             Prints an HashMap as a list of index + key + value |
| `static boolean` | `treeMapsHaveEqualValues(java.util.TreeMap<java.lang.Integer,java.lang.Character> map1, java.util.TreeMap<java.lang.Integer,java.lang.Character> map2)`             Check if two treeMaps have the same collection of values |

**Methods inherited from class java.lang.Object**

`equals, getClass, hashCode, notify, notifyAll, toString, wait, wait, wait`

**Constructor Detail**

### MapUtils

```
public MapUtils()
```


**Method Detail**

### extractMapEntry

```
public static java.util.Map.Entry extractMapEntry(java.util.Map map,
                                                  int i)
```

:   Extracts a random entry (key-value association) from a map

    :   **Parameters:**: `map` - a map **Returns:**: a random (truncated Pareto distributed) entry from the map

---


### fromTwoVectorsToMap

```
public static java.util.TreeMap<java.lang.String,java.lang.String> fromTwoVectorsToMap(java.util.Vector<java.lang.String> keys,
                                                                                       java.util.Vector<java.lang.String> values)
```

:   Creates an hashmap from 2 vectors. The first vector must contain a the
    keys, the second vector the values. The keys and values vectors must have
    the same size.

    :   **Parameters:**: `keys` -: `values` - **Returns:**: an `HashMap`

---


### getDoubleFromHashmap

```
public static double getDoubleFromHashmap(java.util.HashMap hashMap,
                                          java.lang.String hashMapKey)
```

:   Gets a double from a hashMap

---


### getFirstValueOccurrence

```
public static java.util.ArrayList<java.lang.String> getFirstValueOccurrence(java.util.Map<java.lang.String,java.lang.String> map,
                                                                            java.lang.String value)
```

:   Get all the keys that have the given value in a Map\

    :   **Parameters:**: `map` - a map: `value` - a hash map value **Returns:**: the keys

---


### getIndexedMap

```
public static java.util.Map<java.lang.Integer,java.util.Map.Entry> getIndexedMap(java.lang.Object[] mapEntries)
```

:   Get a map with entries composed of: an integer id as key and an entry as
    value

    :   **Parameters:**: `mapEntries` - an array of Entry objects **Returns:**: a map with entries composed of: an integer id as key and an entry as value

---


### mapToString

```
public static java.lang.String mapToString(java.util.Map map)
```

:   Creates a string representation of a map, as index,tab,key,tab,value

    :   **Returns:**: a string representation of the map

---


### printMap

```
public static void printMap(java.util.Map map)
```

:   Prints an HashMap as a list of index + key + value

    :   **Parameters:**: `map` -

---


### treeMapsHaveEqualValues

```
public static boolean treeMapsHaveEqualValues(java.util.TreeMap<java.lang.Integer,java.lang.Character> map1,
                                              java.util.TreeMap<java.lang.Integer,java.lang.Character> map2)
```

:   Check if two treeMaps have the same collection of values

    :   **Parameters:**: `map1` -: `map2` - **Returns:**: true, if the two treeMaps have the same collection of values


---


|  |  |  |
| --- | --- | --- |
| **Overview** \| **Package** \| **Class** \| **Use** \| **Tree** \| **Deprecated** \| **Index** \| **Help** | |  |
| **PREV CLASS**   **NEXT CLASS** | **FRAMES**    **NO FRAMES**     **All Classes** |  |
| SUMMARY: NESTED \| FIELD \| CONSTR \| METHOD | DETAIL: FIELD \| CONSTR \| METHOD |  |


---
